# Intermittent preventive treatment for children (IPTC) combined with timely home treatment for malaria control

**DOI:** 10.1186/1475-2875-11-S1-P108

**Published:** 2012-10-15

**Authors:** Collins K Ahorlu, Kwadwo A Koram

**Affiliations:** 1 Department of Epidemiology, Noguchi Memorial Institute for Medical Research, University of Ghana, Box LG581, Legon, Ghana

## Background

Malaria is estimated to cause between 300 and 500 million clinical cases with about 700,000 to 1.6 million deaths every year, most of these deaths occur in sub-Sahara Africa. In Ghana malaria accounts for about 32.5% of all Out Patient attendances and about 24.6% of deaths in children under 5 years. The World Health Organization recommended the use of artemisinin based combination therapy (ACT) for the treatment of uncomplicated malaria to provide effective treatment against *P. falciparum* malaria and slow down the spread of drug resistance. Ghana had adopted the use of Artesunate + Amodiaquine for the treatment of uncomplicated malaria in the country since 2005. Malaria control in Ghana, like elsewhere in sub-Sahara Africa, relies on early diagnosis and prompt treatment of suspected cases and the home is where early recognition and in most cases prompt treatment is initiated. However, the current combination therapy is not widely available for home management as a result of the fear that making these drugs available may lead to abuse and therefore lead to the emergence of *Plasmodium falciparum* resistance to these drugs. Intermittent preventive treatment (IPT) has now been accepted as an important component of the malaria control strategy but has not been implemented in combination with timely home management to measure their impact on malaria prevalence in a target population.

## Methods

This paper reports finding from a two year implementation of a combined intermittent preventive treatment for children (IPTC) and timely home management of malaria using Artesunate + Amodiaquine. All children aged six to 60 months received home-based delivery of intermittent preventive treatment using Amodiaquine + Artesunate, delivered at home by community assistants every four months (six times in 24 months). Malaria parasite prevalence surveys were conducted before the first and four months after the third and sixth IPTC rounds to serve as baseline, year-one and year-two evaluations.

## Results

Results showed a significant reduction in malaria prevalence from 25% at baseline to 1% at year-two evaluation. At baseline, 13.8% of the children were febrile (axilary temperature of ≥37.5°C) compared to 2.2% at year-one-evaluation while about 2.0% were febrile at year-two-evaluation. The improved access to ACT dugs for the treatment of suspected malaria in children aged six to 60 months could be one of the ways to achieve the Abuja target of getting 60% of under five suspected malaria cases into treatment within 24 hours of symptom onset, which was not achieved by 2005 as was targeted.

## Conclusion

IPTc combined with timely treatment at home could be an effective tool for malaria control in sub-Saharan Africa, especially in difficult to reach areas and this must be looked favourably by policy makers if malaria elimination should be a reality in the nearest future. If policy makers may be bold to initiate policy to allow adults to participate in IPT at least once or twice in a year, this could further accelerate the reduction in malaria prevalence to a level that will reduce the public health burden of malaria.

